# Closed loop insulin delivery–Opportunities and limitations

**DOI:** 10.1111/1753-0407.13490

**Published:** 2023-10-19

**Authors:** Ram Weiss

**Affiliations:** ^1^ Department of Pediatrics Ruth Children's Hospital, Rambam Medical Center and the Bruce Rappaport School of Medicine at the Technion Haifa Israel

The management of type 1 diabetes (T1DM) involves frequent blood glucose monitoring and continuous adjustment of insulin delivery in order to maintain blood glucose within a narrow range as close as possible to normoglycemia. Two major advancements in the field revolutionized the treatment of T1DM in last decades: first, continuous delivery of rapid acting insulin via a continuous infusion pump allows imitating natural insulin delivery much better than multiple daily injections; second—continuous real‐time glucose monitoring devices provide information regarding glucose trends as well as real‐time alarms indicating low or high glucose levels. Although both of these advancements provide major insights and capabilities for improving metabolic control, despite the rise of their use, their impact on glycemic outcomes was rather disappointing. Based on T1DM Exchange data^1^—mean glycated hemoglobin (HbA1c) remained stable among adults with T1DM and even slightly deteriorated among teenagers as the use of these devices grew. A potential explanation for this observation is that although adding information and improving accuracy of insulin delivery—the use of such devices actually increased treatment burden by adding more daily treatment dilemmas and alarms that may be disturbing to some patients.

The major advancement of recent years is the hybrid closed loop (HCL) automated insulin delivery (AID) system, which consists of a combination of a real‐time continuous glucose monitor (CGM) feeding glucose data to an algorithm that then adjusts the delivery of insulin via the insulin pump based on ambient glycemia. The system is considered a “hybrid” closed loop because the patients are still required to provide a “meal declaration,” which is usually an estimation of the amount of carbohydrates to be consumed. AID systems have been gaining popularity across the globe and radically changed the landscape of T1DM management. There are now several approved commercial systems, each using different pumps, CGMs, and algorithms. There is also widespread use of noncommercial systems, known as do‐it‐yourself (DIY) systems, leveraging open source algorithms with commercially available CGMs and pumps.^2^ DIY systems are widely used due to their simple and wide accessibility, among other reasons.^3^

All commercial systems underwent pivotal clinical trials for regulation and proof of efficacy and all showed very similar short‐term effects on glycemic outcomes. Specifically, the vast majority of patients on commercial HCL systems who participated in these trials spent more than 70% of the time within the desired glycemic range (of 70–180 mg/dL) and achieved an HbA1C of ~7% ± 0.5%. Moreover, <2% of the time was spent at the hypoglycemic range below 70 mg/dL.^4^, ^5^, ^6^ Similar glycemic outcomes were demonstrated for multiple open source DIY systems.^7^ Importantly, several longer observational trials with larger patient populations demonstrated similar results to the shorter pivotal trials.^8^ These studies span a wide age range of patient populations and show an almost uniform positive impact of these systems on glycemic outcomes. Importantly, real life data of users of HCLs from participants across the globe shows very similar glycemic outcomes proving that the positive impact of the systems is independent of cultural, geographic, or health system related factors.^9^, ^10^ Of note, the DIY noncommercial HCLs achieved similar real life glycemic outcomes as the official systems. In addition to promising glycemic outcome results, the main advantage of HCLs is by the reduction of treatment burden. Specifically, these systems achieve a time in range of ~90% during the nighttime (when supposedly the patient is sleeping and not eating). Fear of hypoglycemia, specifically during sleep, is a major barrier to tight glycemic control in patients with T1DM and HCLs provide a major breakthrough in this aspect.^11^

Can novel HCLs algorithms perform better than those already in use? There are attempts to use artificial intelligence (AI) and machine‐learning methodologies to develop patient‐tailored algorithms based on the identification of glycemic patterns in relation to activity levels, specific time windows during the day/week. or additional metabolic factors. For example, the algorithm can learn to identify glycemic excursion patterns that develop during or following specific physical activities and adjust insulin delivery in order to reduce these excursions once such activity is identified using input from additional physiological sensors (such as pulse monitoring, actigraphy etc.). In females, insulin sensitivity and action may be affected by the phase of the menstrual cycle,^12^ which can be identified using physiological measures such as body temperature or patient‐controlled apps. Multiple attempts are being pursued to simplify carbohydrate counting by using image recognition apps in order to provide the algorithm with patient‐independent estimates of the contents of the meal to be consumed.^13^ In addition, there are attempts to provide the algorithms with meal recognition capabilities that will allow complete “closure” of the loop without any patient input to the system.^14^ Clinical trials using a complete “closed loop” have recently been published with a positive proof of concept and promising preliminary results.^15^, ^16^ The question that arises is whether such fine‐tuning elements of the algorithms will provide an additional metabolic glycemic improvement of clinical significance in the face of the multiple technical factors that limit the optimization of HCLs.

Although CGM devices have reached adequate accuracy and precision in order to provide reasonably reliable glycemic data to the algorithm, the main limitation of HCLs remains insulin and its delivery. Since the discovery of insulin over 100 years ago, it is delivered subcutaneously. The subcutaneous absorption rate of rapid acting insulin analogs still fails to match the physiological profile of insulin concentrations in the systemic circulation following a meal. Subcutaneous insulin absorption is a major limitation to HCLs as the time lag between insulin delivery and insulin action is significantly longer in comparison to the rate of carbohydrate absorption from the gastrointestinal tract into the bloodstream.^17^ Moreover, the markedly longer half‐life of subcutaneously administered insulin leaves a long active amount of insulin in the circulation following its delivery. Despite the development of ultra‐short acting insulin, its usage in HCLs did not show a significant advantage in regards to glycemic outcomes.^18^ The relatively slow Pk/Pd profile of subcutaneously delivered insulin is a major barrier to the ability of algorithms using multiple inputs to detect meal initiation and provide an insulin delivery that will be able to prevent an early postprandial glycemic excursion. Importantly, insulin is very sensitive to ambient temperature, whether during the supply chain or during patient handling prior to and during use within the insulin pump.^19^ This may lead to actual insulin delivery that is significantly lower than the algorithm driven amount intended.

In addition to the biochemical properties of the insulin itself, the transdermal catheters of insulin pumps suffer from their own limitations. The maximal plasma concentration (C_max_) and time to reach C_max_ may differ between the days following catheter insertion by up to 25% and 50% respectively.^20^ Moreover, repeated insertion of catheters in the same vicinity, as commonly observed in many patients using insulin pumps, results in local lipohypertrophy that hampers insulin absorption even more.^21^ Similarly, it has been known for several decades that the location of insulin delivery has a strong impact on insulin absorption kinetics. Abdominal catheter insertion usually, but not necessarily, results in a greater insulin concentrations compared to catheter insertion in the thighs. Local temperature of the insulin delivery site is also a major determinant of insulin pharmacokinetics.^22^ The magnitude of these variations imposes significant challenges to algorithms that do not receive direct inputs regarding actual plasma insulin concentrations, but rather rely on delivery history and given predetermined Pk/Pd parameters that might not reflect the dynamic nature of such parameters that is due to biological factors that are neither monitored nor measured. Alternative routes of insulin administration such as intra‐peritoneal delivery may result in improved glycemic control in comparison subcutaneous delivery yet suffer from limitations of their own.^23^

Current HCL insulin delivery systems represent a game‐changing advancement in the care of patients with T1DM. Their performance in regards to glycemic outcomes as of now is far superior to that achieved by standard tools by the vast majority of patients. Thus, the use of HCLs should be considered standard of care for patients with T1DM regardless of their age. In order to further optimize the performance of HCLs—we need to overcome the major technical issues related to insulin and its delivery. The performance of mathematical algorithms is as good as the reliability of the parameters they use. Presently, the algorithms assumptions regarding insulin concentration (based on presumed delivery by the pump) are suboptimal at best due to the aforementioned limitations. In order to overcome the limitations of the current HCL systems—the issues of insulin pharmacokinetics and delivery must be addressed. While novel ultra‐rapid insulins are in development, faster and more reliable insulin delivery routes should be sought. The limitations of the transdermal insulin delivery approach using the present catheters will continue to be a barrier whose impact on glucose excursions is probably much greater than that of potential algorithm fine‐tuning. Using the current insulins and pump catheters will make it challenging to completely “close the loop” and be able to detect early glucose excursions and respond fast enough to prevent significant postprandial hyperglycemia. Innovative algorithms attempting to “close the loop” are being developed and tested clinically and the hope is that the technical barriers discussed here will be overcome while aiming at a near normal glycemia safely.

## AUTHOR CONTRIBUTIONS

The author wrote this paper on his own and is responsible for its content.

## FUNDING INFORMATION

No funding was provided for the writing of this commentary.
